# Supplementary material for: Investigating sensory response to physical discomfort in children with autism spectrum disorder using near-infrared spectroscopy
Source: PLoS One. 2021 Sep 3;16(9):e0257029. doi: 10.1371/journal.pone.0257029 (PMC8415580; doi:10.1371/journal.pone.0257029)
Supplement: Attachment — Submitted filename: Response to Reviewers.docx [file pone.0257029.s001.docx]

**RESPONSE TO REVIEWERS**

**Investigating Sensory Response to Physical Discomfort in Children with Autism Spectrum Disorder using Near-Infrared Spectroscopy**

**PONE-D-21-06009**

**Reviewer #1**

**1. As noted by the authors, the effects of medication on these children were not controlled for or reported. This may have a significant impact of the perception of pain and the attentional abilities of the child.**

We have added these potential effects to our discussion regarding medication.

Added text:

*Limitations*

*ASD Population*

*It should be noted that medications can potentially affect hemodynamic and/or cortical activity, as well as pain perception and attentional abilities.*

**2. The authors site the work of Dunn, Schoen, and Miller demonstrating an understanding of sensory processing disorder and its classifications. There is no description of the sensory processing disorder diagnosis or severity in this manuscript. Children with over- and under-responsive types of SPD will naturally respond differently to stimuli.**

Unfortunately, we do not have this information specifically regarding sensory processing diagnoses or severity for the participants of this study. This has been added as a limitation and consideration for future work.

Added text:

*Limitations*

*ASD population representation*

*It should also be noted that sensory processing disorders can also present in the general population. Participants should be screened explicitly for sensory processing disorders and severity in future work, and considerations of this subgroup ought to be considered.*

**3. Since children with SPD may occur in the general population, often without co-morbid diagnoses, control group children should have been screened for SPD as well.
Suggestions to consider in future studies**

This information has been added to the manuscript.

Added text:

*Limitations*

*ASD population representation*

*It should also be noted that sensory processing disorders can also in the general population. Participants should be screened explicitly for sensory processing disorders and severity in future work, and considerations of this subgroup ought to be considered.*

**4. Children with SPD have altered reactions to sensory habituation based on the type of stimuli and the presentation timing of the stimuli. Single modal stimulation often has a muted response, while multi-modal stimuli has a delayed response that is typically additive across trials. See the Sensory Challenge Protocol studies from Schaaf and Miller.**

We have included this information and reference to the work of Schaaf and Miller in the manuscript.

Added text:

*Assessment of physical pain or discomfort*

*However, Sensory Processing Disorder and an atypical response to ordinary stimuli is believed to stem from brain rather than peripheral nervous system dysfunction (10,11). Specifically, atypical sensory modulation is believed to stem from the brain’s inability to appropriately regulate the received sensory information to produce a suitable output (5).*

*Aberrant sensory processing in the ASD population*

*Individuals with sensory processing disorders can have altered reactions to sensory stimuli that depend on the type of stimulus, its timing, and the number of sensory systems stimulated at once (11). Thus, a larger heterogenous sample of the population and different types of stimuli ought to be considered in future work to evaluate generalizability of these findings. Capturing the heterogeneity in ASD would yield greater understanding of the neurobiological underpinnings of atypical sensory processing and pain perception.*

**5. Another consideration would be the effect of decreased vagal tone on the physiologic response to pain. Typically children with SPD demonstrate lower heart rate at rest. When in pain, most individuals will show an increase in HR due to parasympathetic responses to noxious stimuli. By measuring HR while looking at the blood flow to the brain, there may be a way to determine physiologic response vs. perception of pain.**

Consideration of vagal tone to evaluate sensory processing in our paradigm has been added to the manuscript.

Added text

*Clinical relevance of findings and expanded applications*

*Additionally, consideration of other autonomic measurements, such as heart rate, vagal tone, and electrodermal activity, in conjunction with cortical activity may better capture the physiological response to sensory stimuli (53,54). Our focus was on assessing the utility of NIRS to measure a response. However, autonomic measurements have shown to be reliable indicators of hyper or hypo sensitivity and help separate physiological pain from perception. A multi-modal analysis could be considered in future work.*

**6. Electrodermal measurements have also been shown to be a reliable indicator of sensory over- or under-arousal status.**

Consideration of electrodermal activity to evaluate sensory processing in our paradigm has been added to the manuscript.

Added text

*Clinical relevance of findings and expanded applications*

*Additionally, consideration of other autonomic measurements, such as heart rate, vagal tone, and electrodermal activity, in conjunction with cortical activity may better capture the physiological response to sensory stimuli (53,54). Our focus was on assessing the utility of NIRS to measure a response. However, autonomic measurements have shown to be reliable indicators of hyper or hypo sensitivity and help separate physiological pain from perception. A multi-modal analysis could be considered in future work.*

**Overall, I think this is a good article. I would add data on the SPD sub-type with which the children present, or list it as a limitation of this study.**

**Reviewer #2**

**1. While well written, the manuscript is too long. Specifically, the introduction and discussion should be abbreviated to include only the most pertinent information. Most of the background information should be made more concise. In general, any claim should be supported by a relevant citation. The first several paragraphs of the introduction have many claims without citation and are quite verbose. Recommend making the introduction much more concise.**

We have edited the manuscript to shorten it and remove unnecessary information.

**2. A full discussion of the subject inclusion and exclusion is recommended. This should include both demographic inclusion criteria as well as clinical. Furthermore, the recruitment strategy should be discussed. Also, if any compensation was involved. Please describe the consenting process and other ethical considerations, including the parental roles.**

We have included additional information regarding recruitment processes, the consent process, parental/guardian involvement and the compensation.

Added text

# *Methods*

## *Participants*

*Twenty-two male participants were recruited for this study through Holland Bloorview Kids Rehabilitation Hospital (Toronto, Ontario, Canada) and the Province of Ontario Developmental (POND) Network. Participants in the POND Network who agreed to receive study recruitment emails received a study flyer via email and were invited to contact the study’s research coordinator for more information if interested. Fifteen participants were individuals with ASD, and 7 were typically-developing (TD) controls. All participants were between the ages of 10-15 years and met the following inclusion/exclusion criteria: normal or corrected to normal vision, able to understand and communicate in English, no history of chronic pain, hypertension or heart conditions, and no hand abrasions.*

*Ethics approval was obtained from Holland Bloorview Kids Rehabilitation Hospital, and informed written consent was obtained from each participant prior to study participation. Participants were given a description of the study and their understanding of the study was assessed through a series of questions. Upon successfully demonstrating their understanding of the study, and if they were still willing to participate, participants and their parent or guardian signed the consent form. As a token of appreciation for their participation, participants were given a gift card (regardless of successful study completion). A parents or guardian was present during the consent process, but not during the experimental protocol.*

**3. This reviewer questions the decision to include the “excluded” subjects in the analysis. Including these subjects, and also analyzing the cohort without them leads to a confusing description of the results. Furthermore, table 2 is quite confusion with these two groups described (with and without usable NIR data). Recommend only reporting results on subjects with a complete dataset.**

We have modified the statistical results presented to only report on subjects with a complete dataset. Table 2 has also been updated.

Added text:

*Results*

*Results are presented for the remaining 10 participants in the ASD group.*

*Participant characteristics*

*The two participant groups were not significantly different with respect to age (p = 0.76, t_16_ = -0.309), but differed significantly with respect to IQ ( p = 0.01, t_15_ = -3.23).*

*Subjective pain ratings*

The pain ratings were significantly different between the two task types (*p* < 0.01, F_70_ = 241.663) but did not different significantly between the two study groups (*p =* 0.628, F_14_ = 0.245).

*Pain/discomfort tolerance*

Statistical analysis of the cold-water trial durations showed no significant differences were observed between the two groups or across the three trials (group: *p* = 0.09, F_45_ = 7.483; trial: *p* = 0.406, F_31_ = 0.929).

**4. Table 2 should also include p values.**

Table 2 has been updated to include p-values.

Updated table:

| Demographic information |  | **All**  **(N = 17)** | **TD**  **(N = 7)** | **ASD**  **(N = 10)** | **p-value** |
| --- | --- | --- | --- | --- | --- |
|  | Age (years) | 12.5 ± 2.0 | 12.9± 1.8 | 12.6 ± 2.2 | age: *p = 0.76* |
|  | Handedness | 1L, 16 R | 0L, 7R | 1L, 9R |  |
|  | Full Scale IQ | 97.3 ± 20.5 | 113.2 ± 12.9* | 87.8 ± 18.3 | IQ:  *p = 0.01* |
| Subjective Pain Ratings |  | **All**  **(N = 16)** | **TD**  **(N = 7)** | **ASD**  **(N = 9)** |  |
|  | Tepid water | 0.21 ± 0.6 | 0.33 ± 0.9 | 0.11 ± 0.3 | groups: *p =* 0.628 |
|  | Cold water | 5.88 ± 2.9 | 6.17 ± 1.8 | 5.65 + 3.5 | task: *p* < 0.01 |
| Pain tolerance (duration of trial) |  | **All**  **(N = 17)** | **TD**  **(N = 7)** | **ASD**  **(N = 10)** |  |
|  | Tepid water (s) | 62.68 ± 5.9 | 64.32 ± 2.8 | 61.58 ± 7.3 | groups: *p* = 0.09 |
|  | Cold water (s) | 127.38 ± 68.9 | 166.30 ± 41.5 | 108.8 ± 73.7 | trials*: p* = 0.406 |

**5. The results section should report on the data only. Further description of methods should be placed in the “methods” section, including the section starting on page 15 line 354**

The manuscript has been edited such that the results section only includes data, and the methods have been moved to the methods section.

Added text:

*Feature extraction*

*Because participants dictated the duration of the cold stimulus trials, the length of the trials varied across participants and task repetition. All control trials were 60s long, while the stimulus trials were up to 180s. To compare the evolution of the hemodynamic response between trial types, trial duration ought to be consistent. Thus, two different comparisons of brain activity were considered for statistical analysis of functional brain measurements: i) maximum [HbO] across the full task intervals and ii) maximum [HbO] within the first 60s of the task intervals.*
