# Supplementary material for: Investigating sensory response to physical discomfort in children with autism spectrum disorder using near-infrared spectroscopy
Source: PLoS One. 2021 Sep 3;16(9):e0257029. doi: 10.1371/journal.pone.0257029 (PMC8415580; doi:10.1371/journal.pone.0257029)
Supplement: Attachment — Submitted filename: Response_July2021.docx [file pone.0257029.s002.docx]

**Response - Investigating Sensory Response to Physical Discomfort in Children with Autism Spectrum Disorder using Near-Infrared Spectroscopy**

Reviewer #3: The authors explored the objective pain assessment tools in ASD by using NIRS and achieved some results. This study was well written after revision. There were some concerns about the methods as follow:

1. The effects of using psychotropic drugs and cognitive level in ASD children should be considered in this study. The IQ level differed significantly between 2 groups, so the authors should discuss the influence of IQ level as a confounding factor.

Regarding the potential effect of medication on the hemodynamic response, this limitation was already addressing in the *Limitation* section on page 21, Ln 496. Regarding IQ, while the two groups differed significantly in IQ, the task did not have a significant cognitive component. As addressed in section *Sample Size*, previous work has shown that cognitive level does not significantly affect sensory reactivity. The text has been modified to clarify this.

*Added text:*

*Sample Size*

Additionally, study groups were not matched for IQ in this work. Although this is a potential confounding factor, it has been found that cognitive level (IQ) or overall developmental level is not related to abnormal sensory reactivity in children with ASD^2^.

2. The authors expressed that self-reporting of pain can be difficult in ASD children and "no history of chronic pain" was one of the inclusion/exclusion criteria. How to confirm "no history of chronic pain" in ASD children？

“No chronic history of pain” was confirmed by the participant and their caregiver during the consent process.

*Added text:*

*Method*

*Participants*

It was also confirmed that participants met the inclusion/exclusion criterion during the consent process and were eligible to participate. Upon successfully demonstrating their understanding of the study and confirming eligibly criterion were met, and if they were still willing to participate, participants and their parent or guardian signed the consent form.
